# Environmental and ecological factors affecting tick infestation in wild birds of the Americas

**DOI:** 10.1007/s00436-024-08246-6

**Published:** 2024-06-26

**Authors:** Ana Busi, Estefani T. Martínez-Sánchez, Johnathan Alvarez-Londoño, Fredy A. Rivera-Páez, Héctor E. Ramírez-Chaves, Francisco E. Fontúrbel, Gabriel J. Castaño-Villa

**Affiliations:** 1 Grupo de Investigación en Genética, Biodiversidad y Manejo de Ecosistemas (GEBIOME), Departamento de Ciencias Biológicas, Facultad de Ciencias Exactas y Naturales, Universidad de Caldas, Calle 65 No. 26-10, 170004 Manizales, Caldas Colombia; 2 Grupo de Investigación en Ecosistemas Tropicales, Facultad de Ciencias Exactas y Naturales, Universidad de Caldas, Calle 65 No. 26-10, 170004 Manizales, Caldas Colombia; 3 Doctorado en Ciencias-Agrarias, Facultad de Ciencias Agropecuarias, Universidad de Caldas, Calle 64B No. 25-65, 170004 Manizales, Caldas Colombia; 4 Doctorado en Ciencias-Biología, Facultad de Ciencias Exactas y Naturales, Universidad de Caldas, Calle 65 No. 26-10, 170004 Manizales, Caldas Colombia; 5 Facultad de Ciencias Exactas y Naturales, Maestría en Ciencias Biológicas, Universidad de Caldas, Calle 65 No. 26-10, 170004 Manizales, Caldas Colombia; 6 Centro de Museos, Museo de Historia Natural, Universidad de Caldas, Calle 58 No. 21-50, 170004 Manizales, Caldas Colombia; 7 Instituto de Biología, Facultad de Ciencias, Pontificia Universidad Católica de Valparaíso, 2373223 Valparaíso, Chile; 8 Grupo de Investigación en Genética, Biodiversidad y Manejo de Ecosistemas (GEBIOME), Facultad de Ciencias Agropecuarias, Universidad de Caldas, Calle 64B No. 25-65, 170004 Manizales, Caldas Colombia

**Keywords:** Aves, Bioclimatic variables, Dilution effect, Hard ticks, Parasite-host interaction

## Abstract

The Americas hold the greatest bird diversity worldwide. Likewise, ectoparasite diversity is remarkable, including ticks of the Argasidae and Ixodidae families – commonly associated with birds. Considering that ticks have potential health implications for humans, animals, and ecosystems, we conducted a systematic review to evaluate the effects of bioclimatic, geographic variables, and bird species richness on tick infestation on wild birds across the Americas. We identified 72 articles that met our inclusion criteria and provided data on tick prevalence in wild birds. Using Generalized Additive Models, we assessed the effect of environmental factors, such as habitat type, climatic conditions, bird species richness, and geographic location, on tick infestation. Our findings show that most bird infestation case studies involved immature ticks, such as larvae or nymphs, while adult ticks represented only 13% of case studies. We found birds infested by ticks of the genera *Amblyomma* (68%), *Ixodes* (22%), *Haemaphysalis* (5%), *Dermacentor* (1%), and *Rhipicephalus* (0.8%) in twelve countries across the Americas. Our findings revealed that temperature variation and bird species richness were negatively associated with tick infestation, which also varied with geographic location, increasing in mid-latitudes but declining in extreme latitudes. Our results highlight the importance of understanding how environmental and bird community factors influence tick infestation in wild birds across the Americas and the dynamics of tick-borne diseases and their impact on biodiversity.

**Supplementary Information:**

The online version contains supplementary material available at 10.1007/s00436-024-08246-6.

## Introduction

The Americas have the greatest bird diversity worldwide (Orme et al. 2006), which explains the large diversity of ectoparasites associated with wild birds. Among the ectoparasites related to birds, ticks of the families Argasidae (*⁓* 108 spp.) and Ixodidae (*⁓* 137 spp.) are also diverse in terms of number of species (Nava et al. 2017; Dantas-Torres et al. 2019; Guglielmone et al. 2021; Instituto Nacional de Tecnología Agropecuaria [INTA] 2022; Guglielmone et al. 2023). In the Americas, wild birds are hosts and dispersers of larvae, nymph, and adult ticks of different genera of Argasidae (e.g., *Argas* and *Ornithodoros*) and Ixodidae (e.g., *Ixodes, Haemaphysalis, Amblyomma, Dermacentor*, and *Rhipicephalus*) (Morshed et al. 2005; Guglielmone et al. 2014; Nava et al. 2017; Gomez-Puerta et al. 2020). These avian hosts may have multiple interactions with different tick species, highlighting the complex ecological interactions between birds and ticks.

Tick host specificity can greatly vary among definitive and intermediate hosts (Nava and Guglielmone 2013; Esser et al. 2016). Immature ticks (larvae and nymphs) exhibit a generalist and opportunistic feeding behavior, parasitizing a wide range of vertebrate hosts, including many avian species. In contrast, most adult ticks are specialized to specific mammalian hosts (Nava and Guglielmone 2013; Esser et al. 2016; Fecchio et al. 2020a). Moreover, some nidicolous ticks, such as *Argas persicu*s (Oken, 1818), *Ixodes auritulus* (Neumann, 1904), *Ixodes brunneus* (Koch, 1844), or *Ixodes uriae* (White, 1852), have a high degree of host specificity, relying on birds for their entire life cycle and live predominantly in the nests or burrows of their avian hosts (Sonenshine and Roe 2013; Guglielmone et al. 2014, 2023; Nava et al. 2017). In contrast, non-nidicolous tick species, such as *Amblyomma longirostre* (Koch, 1844) or *Amblyomma nodosum* (Neumann, 1899), wait for their avian hosts in emergent vegetation and only parasitize birds in their immature stage (Sonenshine and Roe 2013; Guglielmone et al. 2014, 2023). Additionally, generalist tick species like *Amblyomma calcaratum* (Neumann, 1899) or *Ixodes pacificus* (Cooley and Kohls 1943) can parasitize several orders of birds and mammals during their immature stages (Guglielmone et al. 2014, 2023). On the other hand, in adult stages, ticks like *A. longirostr*e, *A. nodosum*, or *A. calcaratum* typically display a certain degree of specialization, depending on mammal hosts of the families Erethizontidae (New World porcupines) or Myrmecophagidae (anteaters) (e.g., *Coendou prehensilis* (Linnaeus, 1758), *Coendou quichua* (Thomas, 1899), *Coendou spinosus* (F. Cuvier, 1823), *Tamandua tetradactyla* (Linnaeus, 1758) or *Tamandua mexicana* (Saussure, 1860)) (Guglielmone et al. 2014). This biological diversity in feeding habits and host specificity underscores the complexity of tick interactions. Understanding this is crucial, as some tick species are vectors of pathogenic organisms such as bacteria (e.g., *Rickettsia*, *Anaplasma*, or *Borrelia*), protozoa (e.g., *Babesia* or *Theileria*), nematodes (e.g., *Ackertia* or *Monanema*), and viruses (e.g., Crimean-Congo hemorrhagic fever virus or tick-borne encephalitis virus) that negatively affect human welfare, domestic and wild animals health, and the economy (Jongejan and Uilenberg 2004; Estrada-Peña et al. 2012; Boulanger et al. 2019; Tokarz and Lipkin 2020; Erkyihun and Alemayehu 2022).

Several studies have examined tick infestation in wild birds, but these have been relatively few and scattered across the Americas. Wild birds in the Americas display varying levels of tick infestation. For example, in Neotropics, tick infestation rates range from 8 to 28%, while in temperate regions, values range from 4 to 70% (Klich et al. 1996; Morshed et al. 2005; Miller et al. 2016; Domínguez et al. 2019). It has been broadly suggested that tick infestation risk in birds is mediated by environmental factors such as geographic location (e.g., latitude), climatic conditions (e.g., temperature and precipitation), and ecological factors such as habitat type and disturbance (e.g., fragmentation), and bird species richness (Lindgren et al. 2000; Ogrzewalska et al. 2011; Jore et al. 2014; Fecchio et al. 2021a; Lilly et al. 2022). In this context, a comprehensive knowledge of the environmental and ecological factors influencing tick infestation in wild birds across the Americas is crucial for understanding the dynamics of tick-borne parasite transmission in the context of potential climate change scenarios, habitat disturbance, and biodiversity loss.

We conducted a systematic review of the available literature to determine the factors (e.g., weather or habitat degradation) and bird richness that determine the infestation of wild birds in the Americas. Given that the prevalence of tick-infested birds changes with temperature and humidity, habitat type, and host diversity (LoGiudice et al. 2003; Oorebeek and Kleindorfer 2008; Ogrzewalska et al. 2011; Fecchio et al. 2021a), we hypothesized that tick infestation in wild birds will be positively correlated with habitat degradation, temperature, and precipitation because these conditions seem to be needed for establishment, development, and host-seeking of ticks.

## Methods

### Literature survey and data inclusion criteria

**Fig. 1:**
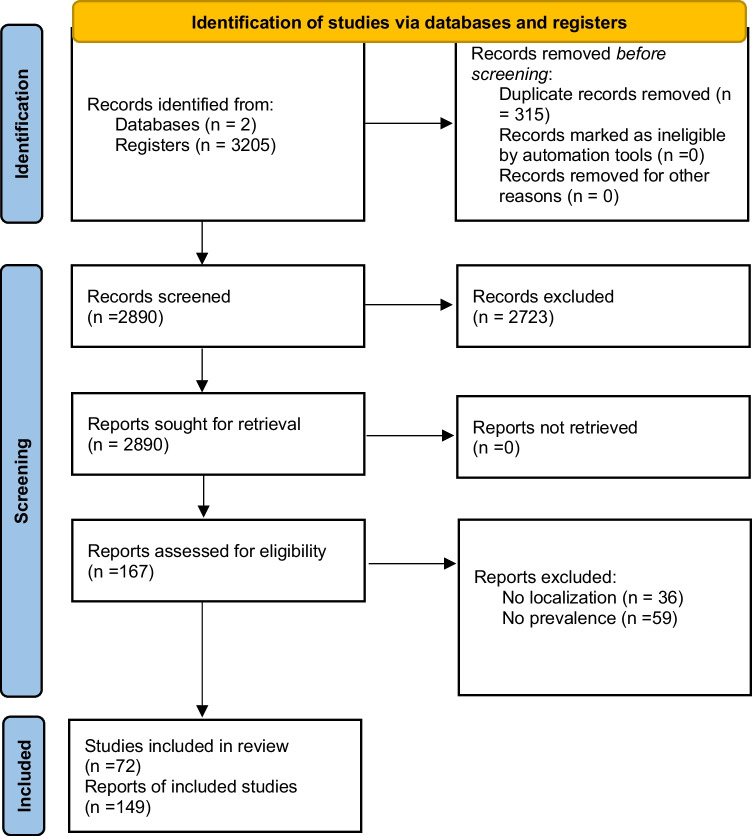
Preferred reporting items for systematic reviews and meta-analysis (PRISMA) flowchart, summarizing the sequence of information selection followed

We conducted a literature search using the Web of Science and Scopus databases (January 1960 to December 2022), using the search terms “bird*” OR “avian*” AND “tick*” in the title, abstract, and keywords. To ensure a standard of quality of results and study replicability, we limited our search to peer-reviewed articles in English, excluding reviews or other documents (e.g., books, theses, technical reports, or institutional dossiers) that may contain duplicate information from articles (Bohada-Murillo et al. 2021). The literature review followed the methodology proposed in the PRISMA statement (Page et al. 2021). The initial search yielded 3205 articles, which were reduced to 2890 after eliminating duplicates. Then, we reduced them to 167 articles after discarding those that did not contain information about tick-infested wild bird communities. The selected articles were thoroughly reviewed to determine if they met the following inclusion criteria: (1) reported prevalence of tick infestation in wild birds or information allowing its calculation, (2) georeferenced location or detailed description of the sample area that allows its location. Inclusion criteria assessment was performed by the same person (AB) to avoid a potential inter-observer bias. We identified 72 articles that met our inclusion criteria and provided data on tick prevalence in wild birds. Nineteen articles present prevalence data for multiple localities, resulting in 149 case studies (i.e., reports of tick infestations from unique study locations), each one representing an observation of tick prevalence in wild birds within specific localities (Fig. 1). Of the 72 included articles, 261 case studies focused on ticks identified at the genus level, infesting wild birds: 179 of *Amblyomma*, 57 of *Ixodes*, 14 of *Haemaphysalis*, and 11 of other genera (Table 1). These case studies represent a subset categorized by tick genus out of the total observations, each corresponding to a specific observation of tick prevalence within a particular tick genus and locality.

**Table 1:** Number of case studies of tick infestation in wild birds, categorized by tick genera and life stage

Genera	Number of cases (%)	Number of cases of infestation				
		Immature			Adult	Unknow
		Larve	Nymph	Immature^a^		
*Ixodes*	57 (21.8)	18	14	22	11	4
*Haemaphysalis*	14 (5.4)	7	5	4	2	1
*Amblyomma*	179 (68.6)	82	100	27	2	1
*Dermacentor*	3 (1.1)	1	1	0	1	1
*Rhipicephalus*	2 (0.8)	1	1	0	1	0
Unidentified	6 (2.3)	1	0	4	1	0

^a^ Case studies where the larval or nymphal life stage is not distinguishable. The term "Unknown" is used for case studies where the tick's life stage is not reported

### Data extraction

From each article, we extracted tick infestation prevalence information (including ticks in any life stage) distinguishing larva, nymph, immature (i.e., larva and nymph), or adult stages along with tick genera. Prevalence represents the proportion of infested birds to the total birds examined (i.e., *prevalence* = number of infested birds/number of examined birds × 100) (e.g., Cardona-Romero et al. 2020; Dumas et al. 2022). In addition, we extracted locality information (country and habitat type), geographic location (i.e., latitude and longitude coordinates and elevation), and bird species richness (i.e., the number of bird species examined at each locality, de Angeli et al. 2021). When the articles did not explicitly provide the coordinates, we employed a systematic approach to estimate them. If the articles included specific details such as landmarks, geographic features, and mentions of towns or forest reserves, we utilized Google Earth Pro 7.3.4 software to estimate the coordinates. In addition, if the habitat information was not detailed in the article, we used the 'historical imagery' feature to complete the habitat description (Google LLC 2021). This feature allowed us to access images from specific periods corresponding to each article's description. In articles where a single prevalence value was reported for multiple localities within the same region or in proximity, we established one coordinate using ecological criteria to ensure unit homogeneity (Strnad et al. 2017). We obtained bioclimatic information for each locality using the dataset WorldClim 2.1 with a resolution of 2.5 min (Fick and Hijmans 2017). The bioclimatic variables included in our analysis represented temperature and precipitation metrics. Temperature variables included annual mean temperature (BIO1), maximum temperature of the warmest month (BIO5), and minimum temperature of the coldest month (BIO6). Precipitation variables included annual precipitation (BIO12), precipitation of the wettest month (BIO13), and precipitation of the driest month (BIO14). Additionally, we considered the differences between the maximum temperature of the warmest month and the minimum temperature of the coldest month (Delta temperature hereafter) and between the precipitation of the wettest month and the precipitation of the driest month (Delta precipitation hereafter). These variables were selected because they are known to influence the activity and phenology of ticks (Estrada-Peña et al. 2014; Nava et al. 2017). We classified habitat types based on the intensity of agricultural land use as natural (habitats covered by native vegetation with no agricultural use), semi-natural (habitats dominated mainly by natural vegetation but indirectly modified for agricultural activities), or agricultural-semiurban (habitats directly managed for agriculture or located directly adjacent to or within an urban environment) (Flynn et al. 2009).

### Statistical analyses

We evaluated the effects of bioclimatic variables, geographic location, habitat type, and bird richness on the proportion of infested birds (infestation hereafter). Independent variables, such as bioclimatic factors and elevation, were centered to facilitate interpretation in our models (Schielzeth 2010). To avoid multicollinearity, we initially used a correlation matrix to identify and remove highly correlated variables (e.g., *r* >|0.75|). Subsequently, we applied the Variance Inflation Factor (VIF) to the remaining variables, refining our model selection (VIF > 5 indicates high multicollinearity) (James et al. 2021). We fitted Generalized Additive Models (GAM) with a quasi-binomial error distribution and a logit link function, using the proportion of infested birds as the response variable (Wood 2017). We included bird richness, elevation, and the bioclimatic variables (BIO6 and BIO13 or Delta temperature and Delta precipitation, depending on model configuration) as linear predictor variables. Habitat type was treated as a categorical variable (natural, semi-natural, or agricultural-semiurban), which was included as a fixed factor in our models to compare tick infestation across the habitat categories explicitly. To account for the spatial effects on bird infestation, coordinates were added as smooth terms in each model, using both latitude and longitude (spatial location hereafter), or only latitude, to determine the most appropriate spatial model representation (Hunsicker et al. 2016; Wood 2017). For detailed model specifications and retained variables in the best models ranked using the Akaike Information Criterion (AIC), see Table S1. We assessed the degree of non-linearity of the smooth terms in our models by calculating the effective degrees of freedom (*edf*); an *edf* value of 1 suggests that the relationship between the predictor and the response is almost linear, while *edf* value between 1 and 2 suggests a weakly non-linear relationship, an *edf* value greater than 2 indicates a non-linear relationship (Wood 2017). Separate models were fitted for the wild bird infestation by ticks according to their life stages: Model 1 (included adult and immature ticks), Model 2 (adult ticks), Model 3 (immature ticks), Model 4 (nymphs ticks), and Model 5 (larvae ticks). Additionally, we fitted four models for *Amblyomma* tick infestation according to life stages: Model 6 (adult and immature *Amblyomma* ticks), Model 7 (immatures *Amblyomma* ticks), Model 8 (nymphs *Amblyomma* ticks), and Model 9 (larvae *Amblyomma* ticks). We only develop models for the genus *Amblyomma* (68% of the case studies in the dataset) due to the small number of case studies for the other genera (Table 1). After model selection procedures, we conducted a 'least square means' analysis to perform pairwise comparisons among habitat types (natural, semi-natural, or agricultural-semiurban) regarding bird infestation proportion. All analyses were performed using “mgcv” (Wood 2011) and “lsmeans” (Lenth 2016) packages in R version 4.3.3 (R Core Team 2024).

## Results

### Tick infestation of bird communities in the Americas

Of the 149 case studies selected in our review, 81% (121) reported tick infestations, and 69% came from studies conducted in tropical regions of the Americas (mainly in Brazil, 32%) between 2000 and 2022. Out of all the case studies of bird infestation, 35% documented larvae and 34% nymphs. Immature ticks were reported in 33% of the case studies (i.e., those in which the larval and nymphal stages could not separated). Adult ticks were only present in 13% of the case studies. Likewise, 57% of the infestation case studies were recorded in natural habitats, followed by seminatural habitats (22%) and agriculture-semiurban habitats (21%). The studies identified birds infested by ticks of the genera *Ixodes, Haemaphysalis, Amblyomma, Dermacentor*, and *Rhipicephalus* in twelve American countries. Of the 261 case studies of tick genera recorded, *Amblyomma* was the most common (68%), followed by *Ixodes* (22%) and *Haemaphysalis* (5%) (Table 1). Of the 179 case studies involving bird infestations by *Amblyomma* ticks, 85% occurred in tropical regions, and 57% occurred in natural habitats. The *Amblyomma* tick species most associated with birds were *A. longirostre* (21%), *A. nodosum* (15%), and *A. calcaratum* (8%).

Across the reviewed studies, a total of 2253 bird individuals, representing 570 species, 58 families, and 18 orders, were reported to be infested by ticks. Among these, the families Thraupidae (12%), Turdidae (11%), and Tyrannidae (11%) were most prevalent. Specifically, the species most frequently infested by ticks included *Trichothraupis melanops* (2%) and *Tachyphonus coronatus* (1.5%) within Thraupidae, *Troglodytes aedon* (1.4%, Troglodytidae), and *Catharus ustulatus* (1.3%, Turdidae). The tick genus *Amblyomma* was dominant, accounting for 68% (1530) of the infestations reported, followed by *Ixodes* with 23% (519), and *Haemaphysalis* with 7% (159). The most common tick species identified were *A. longirostre* 18% (406), *A. nodosum* 10% (219), *Ixodes scapularis* 6% (145), and *Haemaphysalis leporispalustris* 6% (138).

### Effects of bioclimatic, geographic variables and bird richness on infestation

Tick infestation in wild birds was associated with climatic conditions, bird species richness, and geographic location. Adult tick infestation was positively correlated with elevation and negatively correlated with temperature variation, precipitation, and richness (Model 2, Table 2). Infestation by nymphal ticks was negatively influenced by temperature variation and elevation (Model 4, Table 2). Moreover, bird species richness negatively affected bird infestation by the genus *Amblyomma* (except for the *Amblyomma* nymph stage) (Model 6 to Model 9, Table 2). A subsequent pairwise comparison in the models revealed significant differences in tick infestation between natural and semi-natural habitats, with the natural habitat showing lower infestation rates across Models 2, 4, and 5 (Table S3, Figure S1). For an overview of each model's predictive variables, please refer to supplementary Tables S1 and S2.

**Table 2:** Summary of the results from generalized additive models (GAMs) assessing the effects of bioclimatic variables, habitat type, bird species richness, and geographical location on the proportion of wild birds infested by ticks

	Intercept		Habitat types				Bioclimatic variables								Bird species richness		Geographical location					
Model			Seminatural Habitat		Natural Habitat		BIO6		Delta temperature		BIO13		Delta precipitation				Elevation		S (Longitude, Latitude)		S (Latitude)	
	Estimate	Std. Error	Estimate	Std. Error	Estimate	Std. Error	Estimate	Std. Error	Estimate	Std. Error	Estimate	Std. Error	Estimate	Std. Error	Estimate	Std. Error	Estimate	Std. Error	edf	Ref.df	edf	Ref.df
Model_1	-2.029**	0.337	0.081	0.378	-0.253	0.334			0.002	0.062			0.001	0.001	< 0.001	0.004	< 0.001	< 0.001	13.9*	18.31		
Model_2	-25.23	1.44	0.849	0.634	-0.885	0.843			-1.28**	0.393			-0.024**	0.004	-0.086**	0.026	0.002**	< 0.001	18.12**	19.64		
Model_3	-2.240**	0.358	0.371	0.941	< -0.001	0.351	0.056	0.072			-0.002	0.002			-0.005	-0.005	< 0.001	< 0.001	14.64*	19.23		
Model_4	-5.034**	0.618	0.736	0.552	-0.153	0.522			-0.144*	0.063			0.001	< 0.001	0.010	0.007	-0.001**	< 0.001			5.202**	6.33
Model_5	-3.902**	0.692	1.064	0.643	0.040	0.610	-0.049	0.062			0.005	0.002			0.003	0.009	< -0.001	< 0.001	7.035	9.576		
Model_6	-2.613**	0.499	0.386	0.441	0.567	0.413			0.076	0.061			0.001	0.001	-0.020**	0.005	< -0.001	< 0.001	8.122	11.27		
Model_7	-2.547**	0.505	0.278	0.453	0.523	0.418			0.085	0.061			0.001	0.002	-0.021**	0.006	< -0.001	< 0.001	7.388	10.11		
Model_8	-4.786**	4.878	0.7689	0.455	0.622	0.430			-0.024	0.035			< 0.001	0.001	-0.009	0.005	< -0.001	< 0.001			1.00**	1.00
Model_9	-3.576**	1.169	0.762	0.625	0.387	0.575			0.025	0.136			< 0.001	0.002	-0.026*	0.012	< -0.001	< 0.001	11.92	15.75		

Intercept, habitat type agricultural-semiurban; BIO6, minimum temperature of the coldest month; Delta temperature, differences between the maximum and the minimum temperature; BIO13, precipitation of the wettest month; Delta precipitation, differences between the maximum and the minimum precipitation. S (longitude, Latitude), smoothed term of the spatial location; S(Latitude), smoothed term of the latitude. Significance codes:'**' 0.01 '*'0.05

**Fig. 2:**
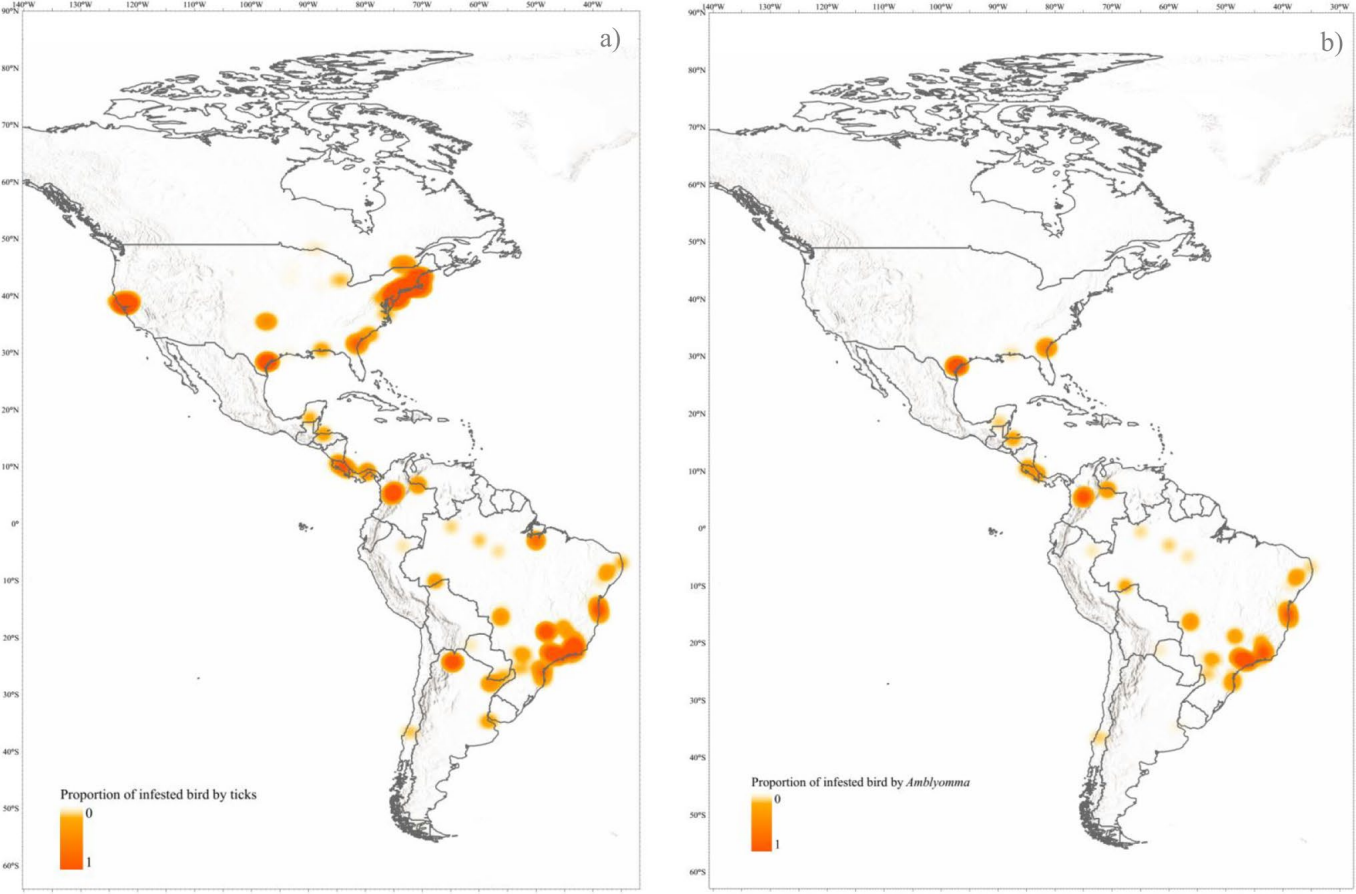
Heat map of the proportion of wild birds infested by ticks in the Americas, according to geographic coordinates **a**) with adult and immature ticks, **b**) with adult and immature ticks of the genus*Amblyomma*

We found that spatial location had a significant influence on tick infestation in wild birds across various life stages, including adults, immatures, and both (Model 1 to 3, Table 2, Fig. 2a, b). Furthermore, latitude significantly affected nymph ticks and *Amblyomma  *nymph ticks (Model 4 and 8, Table 2). High tick infestations in wild birds have been observed in localities from 15° to 28° south latitude and 28° to 45° north latitude, as reported in studies by Beldomenico et al. (2003), Sonenshine and Clifford (1973), and Teel et al. (1998). Lower infestations were found in localities above these latitudes, as reported in studies by Klich et al. (1996), Gonzalez-Acuña et al. (2004), and Cicuttin et al. (2019) (Fig. 2a, b). In the Northern Hemisphere, the USA temperate broadleaf and mixed forests recorded the highest infestation levels. Likewise, the Brazilian Atlantic Forest and Cerrado ecoregions showed the highest infestation levels in the Southern Hemisphere. However, our analysis revealed no significant spatial relationship for larval infestation (Models 5 and 9, Table 2), suggesting that spatial factors were not the determinants of infestation patterns in the tick life stage. We found that bird species richness exhibited a trend across the *Amblyomma* models, except for the *Amblyomma* nymph stage (Model 8, Table 2). Bird infestation shows spatial variability patterns across different localities on the continent in almost half of the models (Model 1 to Model 4, and Model 8, Table 2).

## Discussion

In this study, we found that tick infestation in wild birds in the Americas was related to climatic conditions, bird species richness, and geographic location. Our results showed that tick infestation, especially by *Amblyomma* ticks, in wild birds was negatively associated with bird species richness. This relationship between parasite infestation and community diversity is similar to the 'dilution effect' hypothesis proposed by Keesing and Ostfeld (2021). According to this hypothesis, communities with greater diversity and equity of host species decrease the probability of encounters between ticks and highest-quality hosts (Ostfeld and Keesing 2000; LoGiudice et al. 2008; Civitello et al. 2015). Our estimation of bird species richness served as a proxy of the true richness of bird communities, but it may be influenced by different bird capture methods used in each study (de Angeli et al. 2021). In the case studies reviewed, we observed that lower bird infestation by *Amblyomma* ticks occurred in localities with the highest bird species richness (Ogrzewalska et al. 2008; Maturano et al. 2015; Martinez-Sanchez et al. 2020). Consequently, bird species richness appears to be a determinant of bird infestation in the Americas, which can be affected by habitat disturbance and can lead to changes in infestation patterns (Estades and Temple 1999; Ehlers Smith et al. 2015).

Another factor influencing tick infestation in wild birds in the Americas is the temperature, particularly during the adult and nymphal stages. Climatic variables, especially temperature fluctuations, significantly affect tick distribution, survival, and questing behavior (Cumming 2002; Vail and Smith 2002; Ogden et al. 2004; Berger et al. 2014; Estrada-Peña et al. 2014). Being poikilothermic, ticks exhibit non-linear increases in inter-stadial development rates with rising ambient temperatures (Randolph 2004; Faccini et al. 2021). Under favorable conditions, ticks can remain in questing positions in vegetation for several days. However, they often descend due to increased saturation deficits or atmospheric dryness (Vail and Smith 2002; Randolph 2004; Berger et al. 2014). For example, Oorebeek and Kleindorfer (2008) reported that tick abundance on passerines fluctuates with host availability and climatic conditions, with higher tick populations during months characterized by high humidity, rainfall, and lower temperatures. Additionally, it has been documented that ticks, especially in their immature stages, tend to quest at lower vegetation heights when temperatures are high and relative humidity is low, reducing contact with vertebrate hosts (Lefcort and Durden 1996; Vail and Smith 2002; Randolph 2004; Prusinski et al. 2006; Berger et al. 2014; Portugal et al. 2020). Therefore, temperature variations may be an important determinant of tick infestation patterns in wild birds, affecting their distribution, behavior, and interactions with their hosts.

Tick infestations in wild birds vary with location, suggesting complex ecological interactions in the different localities that influence the bird infestation. We found that bird communities in mid-latitude regions generally have higher proportions of tick infestation, while locations at latitudinal extremes exhibit lower proportions. This result partially supports the notion that the intensity of the parasite-host association increases with latitude (Fecchio et al. 2021b; Zvereva and Kozlov 2021). Environmental changes, especially those related to climate, can alter the ecological niches of ticks and their interaction with host species (Ostfeld and Keesing 2000). It has been proposed that the intensity of association between host and parasites increases at high latitudes due to a variety of factors, such as the presence of a diverse number of parasites or environmental conditions that make parasites more dependent on their hosts (Hawkins 1994; Kamiya et al. 2014; Fecchio et al. 2021b). However, the decrease in infestation at the latitudinal extremes of the Americas could be due to a combination of factors that negatively affect tick density (e.g., mean temperature and saturation deficit) (Diuk-Wasser et al. 2006). Additionally, global climate change effects on biodiversity may impact parasite-host dynamics, suggesting that current infestation patterns may change as ecological conditions change (Lafferty 2009).

Finally, some bird families known to forage in the lower forest strata vegetation (e.g., Thamnophilidae, Furnariidae, Tyrannidae, Pipridae, Troglodytidae, Turdidae, Parulidae, or Thraupidae) seem to be more susceptible to tick infestation (Labruna et al. 2007; Oorebeek and Kleindorfer 2009; Guglielmone et al. 2014; Martinez-Sanchez et al. 2020). This observation may be due to a capture bias, as birds that forage in the lower forest strata vegetation are the ones that are typically captured using traditional methods. Our review is consistent with these findings and shows that families Thraupidae, Turdidae, and Tyrannidae represent the majority of tick-infested birds. Specifically, species such as *T. melanops* and *T. coronatus* within Thraupidae, *T. aedon* within Troglodytidae, and *C. ustulatu*s within Turdidae were commonly infested. This suggests a possible correlation between bird phylogeny and susceptibility to tick infestation, possibly due to shared ecological traits or host phylogenetic conservation, increasing exposure to ticks (Poulin 2007; Barrow et al. 2019; Fecchio et al. 2021b).

Our research identified the environmental and ecological factors influencing tick infestation in wild birds across the Americas. Understanding these factors is critical for assessing the risks associated with tick-borne pathogen transmission (Moller et al. 2013; Fecchio et al. 2020b). We found that climatic conditions are key determinants of tick infestation patterns, as reported in some studies (Cumming 2002; Ogden et al. 2008; Oorebeek and Kleindorfer 2008; Pfaffle et al. 2013; Estrada-Peña and de la Fuente 2014). Furthermore, the inverse relationship between tick infestation and bird species richness highlights the potential role of biodiversity in mitigating disease transmission (LoGiudice et al. 2003; Keesing and Ostfeld 2021). The general patterns described here have implications for disease transmission dynamics, highlighting that environmental and ecological factors modulate the intensity of parasite-host associations and disease risk across different geographic regions.

## Conclusion

Our results show that the prevalence of tick infestation in wild birds in the Americas is related to climatic conditions, bird species richness, and geographic location. Changes in biodiversity resulting from habitat degradation due to climate change could modify the dynamics of tick infestation. In this sense, our results highlight the value of biodiversity as a buffer for parasite infestation in bird communities. Tick infestation in wild birds exhibits complex geographic patterns across different latitudes in the Americas, increasing in mid-latitudes and declining at the extreme latitudes of the continent. Identifying how environmental and wild bird community factors determine tick infestation is crucial to understanding tick-borne disease dynamics and its effects on biodiversity.

### Supplementary Information

Below is the link to the electronic supplementary material.

Supplementary file1 (DOCX 339 KB)

## Data Availability

The data was deposited in Figshare under the reference number 10.6084/m9.figshare.24452050.
