# Supplementary material for: Environmental and ecological factors affecting tick infestation in wild birds of the Americas
Source: Parasitol Res. 2024 Jun 26;123(6):254. doi: 10.1007/s00436-024-08246-6 (PMC11208200; doi:10.1007/s00436-024-08246-6)
Supplement: Supplementary file 1 — Supplementary file1 (DOCX 339 KB) [file 436_2024_8246_MOESM1_ESM.docx]

**Supplementary Material**

**Environmental and ecological factors affecting tick infestation in wild birds of the Americas**

Ana Busi, Estefani T. Martínez-Sánchez, Johnathan Alvarez-Londoño, Fredy A. Rivera-Páez, Héctor E. Ramírez-Chaves, Francisco E. Fontúrbel, Gabriel J. Castaño-Villa

**Table S1.** Summary of predictor variables retained in the nine generalized additive models for wild bird infestation by ticks.

| **Model** | **Habitat** | **BIO6** | **Δ Temp.** | **BIO13** | **Δ Prec.** | **Richness** | **Elevation** | **S (X, Y)** | **S (Y)** |
| --- | --- | --- | --- | --- | --- | --- | --- | --- | --- |
| **Model_1** | X |  | X |  | X | X | X | X |  |
| **Model_2** | X |  | X |  | X | X | X | X |  |
| **Model_3** | X | X |  | X |  | X | X | X |  |
| **Model_4** | X |  | X |  | X | X | X |  | X |
| **Model_5** | X | X |  | X |  | X | X | X |  |
| **Model_6** | X |  | X |  | X | X | X | X |  |
| **Model_7** | X |  | X |  | X | X | X | X |  |
| **Model_8** | X |  | X |  | X | X | X |  | X |
| **Model_9** | X |  | X |  | X | X | X | X |  |

BIO6, minimum temperature of the coldest month; Δ Temp., differences between the maximum and the minimum temperature; BIO13, precipitation of the wettest month; Δ Prec., differences between the maximum and the minimum precipitation; Richness, bird species richness. S (X, Y), smoothed term of the spatial location (longitude and latitude); S (Y), smoothed term of the latitude.

**Table S2**. Results of nine generalized additive models (GAMs) for wild bird infestation by ticks. Below are the detailed results of each of the nine generalized additive models (GAMs) applied to the infestation of wild birds by ticks. Each subsection of the table provides specific results for one model, starting with Model 1 and ending with Model 9. Intercept, Habitat_ agricultural-semiurban; BIO6, minimum temperature of the coldest month; Delta_temperature, differences between the maximum and the minimum temperature; BIO13, precipitation of the wettest month; Delta_precipitation, differences between the maximum and the minimum precipitation; Richness, bird species richness. S (Longitude, Latitude), smoothed term of the spatial location (longitude and latitude); S (Latitude), smoothed term of the latitude.

**Table S2a.** *Model 1,* results of generalized additive model for wild bird infestation by adult and immature ticks, with an adjusted R-squared of 0.328 and 45.5% deviance explained.

| Family: Quasibinomial | | | | | |
| --- | --- | --- | --- | --- | --- |
| Link function: Logit | | | | | |
| **Parametric coefficients:** | **Estimate** | **Std. Error** | ***t* value** | ***p*-value** |  |
| Intercept | -2.029 | 0.337 | -6.011 | <0.001 |  |
| Delta_temperature | 0.002 | 0.062 | 0.046 | 0.963 |  |
| Delta_precipitation | 0.001 | 0.001 | 0.893 | 0.374 |  |
| Elevation | <0.001 | <0.001 | 1.084 | 0.28 |  |
| Habitat_natural | -0.253 | 0.337 | -0.749 | 0.455 |  |
| Habitat_semi-natural | 0.081 | 0.377 | 0.215 | 0.830 |  |
| Richness | <0.001 | 0.004 | 0.025 | 0.980 |  |
| **Approximate significance of smooth terms:** | **edf** | **Ref. df** | **F** | ***p*-value** |  |
| S (Longitude, Latitude) | 13.9 | 18.31 | 1.885 | 0.021* |  |

**Table S2b.** *Model 2,* results of generalized additive model for wild bird infestation by adult ticks, with an adjusted R-squared of 0.842 and 94.4% deviance explained.

| Family: Quasibinomial | | | | | |
| --- | --- | --- | --- | --- | --- |
| Link function: Logit | | | | | |
| **Parametric coefficients:** | **Estimate** | **Std. Error** | **t value** | **p-value** |  |
| Intercept | -0.523 | 0.144 | -1.747 | 0.083 |  |
| Delta_temperature | -0.283 | 0.393 | -3.263 | 0.001 |  |
| Delta_precipitation | -0.024 | 0.003 | -5.572 | <0.001 |  |
| Elevation | 0.002 | <0.001 | 3.550 | <0.001 |  |
| Habitat_natural | -0.885 | 0.843 | -1.050 | 0.296 |  |
| Habitat_semi-natural | 0.849 | 0.634 | 1.341 | 0.182 |  |
| Richness | -0.086 | 0.025 | -3.349 | 0.001 |  |
| **Approximate significance of smooth terms:** | **edf** | **Ref.df** | **F** | ***p*-value** |  |
| S (Longitude, Latitude) | 18.12 | 19.64 | 3.888 | <0.001 |  |

**Table S2c.** *Model 3,* results of generalized additive model for wild bird infestation by immature ticks, with an adjusted R-squared of 0.339 and 48.2% deviance explained.

| Family: Quasibinomial | | | | | |
| --- | --- | --- | --- | --- | --- |
| Link function: Logit | | | | | |
| **Parametric coefficients:** | **Estimate** | **Std. Error** | ***t* value** | ***p*-value** |  |
| Intercept | -2.240 | 0.358 | -6.249 | <0.001 |  |
| BIO6 | 0.053 | 0.072 | 0.743 | 0.459 |  |
| BIO13 | 0.002 | 0.002 | 1.210 | 0.229 |  |
| Elevation | <0.001 | <0.001 | 0.155 | 0.877 |  |
| Habitat_natural | <-0.001 | 0.359 | -0.001 | 1.00 |  |
| Habitat_semi-natural | 0.371 | 0.394 | 0.943 | 0.347 |  |
| Richness | -0.005 | 0.005 | -1.132 | 0.260 |  |
| **Approximate significance of smooth terms:** | **edf** | **Ref.df** | **F** | ***p*-value** |  |
| S (Longitude, Latitude) | 14.64 | 19.23 | 1.746 | 0.036 |  |

**Table S2d.** *Model 4,* results of generalized additive model for wild bird infestation by nymph ticks, with an adjusted R-squared of 0.37 and 43.3% deviance explained.

| Family: Quasibinomial | | | | | |
| --- | --- | --- | --- | --- | --- |
| Link function: Logit | | | | | |
| **Parametric coefficients:** | **Estimate** | **Std. Error** | ***t* value** | ***p*-value** |  |
| Intercept | -5.034 | 0.618 | -8.136 | <0.001 |  |
| Delta_temperature | -0.144 | 0.063 | -2.268 | 0.025 |  |
| Delta_precipitation | 0.001 | 0.002 | 0.682 | 0.497 |  |
| Elevation | -0.001 | <0.001 | -2.743 | 0.007 |  |
| Habitat_natural | -0.153 | 0.522 | -0.295 | 0.768 |  |
| Habitat_semi-natural | 0.736 | 0.552 | 1.332 | 0.186 |  |
| Richness | 0.010 | 0.007 | 1.453 | 0.149 |  |
| **Approximate significance of smooth terms:** | **edf** | **Ref.df** | **F** | ***p*-value** |  |
| S ( Latitude) | 5.202 | 6.332 | 3.108 | 0.007 |  |

**Table S2e.** *Model 5*, results of generalized additive model for wild bird infestation by larvae ticks, with an adjusted R-squared of 0.14 and 37.9% deviance explained.

| Family: Quasibinomial | | | | | |
| --- | --- | --- | --- | --- | --- |
| Link function: Logit | | | | | |
| **Parametric coefficients:** | **Estimate** | **Std. Error** | ***t* value** | ***p*-value** |  |
| Intercept | -3.901 | 0.692 | -5.632 | <0.001 |  |
| BIO6 | -0.049 | 0.062 | -0.798 | 0.427 |  |
| BIO13 | 0.005 | 0.002 | 1.921 | 0.060 |  |
| Elevation | -0.007 | <0.001 | -1.429 | 0.156 |  |
| Habitat_natural | 0.040 | 0.610 | 0.066 | 0.947 |  |
| Habitat_semi-natural | 1.064 | 0.643 | 1.655 | 0.101 |  |
| Richness | 0.003 | 0.009 | 0.391 | 0.696 |  |
| **Approximate significance of smooth terms:** | **edf** | **Ref.df** | **F** | ***p*-value** |  |
| S (Longitude, Latitude) | 7.035 | 9.576 | 1.834 | 0.071 |  |

**Table S2f.** *Model 6,* results of generalized additive model for wild bird infestation by adult and immature *Amblyomma* ticks, with an adjusted R-squared of 0.143 and 29.2% deviance explained.

| Family: Quasibinomial | | | | | |
| --- | --- | --- | --- | --- | --- |
| Link function: Logit | | | | | |
| **Parametric coefficients:** | **Estimate** | **Std. Error** | ***t* value** | ***p*-value** |  |
| Intercept | -0.261 | 0.499 | -5.232 | <0.001 |  |
| Delta_temperature | 0.076 | 0.061 | 1.236 | 0.218 |  |
| Delta_precipitation | 0.001 | 0.001 | 0.792 | 0.429 |  |
| Elevation | <-0.001 | <0.001 | -0.072 | 0.942 |  |
| Habitat_natural | 0.567 | 0.413 | 1.373 | 0.171 |  |
| Habitat_semi-natural | 0.386 | 0.441 | 0.877 | <0.001 |  |
| Richness | -0.020 | 0.005 | -3.487 | <0.001 |  |
| **Approximate significance of smooth terms:** | **edf** | **Ref.df** | **F** | ***p*-value** |  |
| S (Longitude, Latitude) | 8.212 | 11.27 | 1.172 | 0.297 |  |

**Table S2g.** *Model 7,* results of generalized additive model for wild bird infestation by immature *Amblyomma* ticks, with an adjusted R-squared of 0.137 and 28.2% deviance explained.

| Family: Quasibinomial | | | | | |
| --- | --- | --- | --- | --- | --- |
| Link function: Logit | | | | | |
| **Parametric coefficients:** | **Estimate** | **Std. Error** | ***t* value** | ***p*-value** |  |
| Intercept | -2.547 | 0.505 | -5.045 | <0.001 |  |
| Delta_temperature | 0.085 | 0.061 | 1.393 | 0.165 |  |
| Delta_precipitation | 0.001 | 0.002 | 0.965 | 0.335 |  |
| Elevation | <-0.001 | <0.001 | -0.436 | 0.663 |  |
| Habitat_natural | 0.278 | 0.453 | 0.615 | 0.212 |  |
| Habitat_semi-natural | 0.278 | 0.453 | 0.615 | 0.539 |  |
| Richness | -0.021 | 0.006 | -3.396 | <0.001 |  |
| **Approximate significance of smooth terms:** | **edf** | **Ref.df** | **F** | ***p*-value** |  |
| S (Longitude, Latitude) | 7.388 | 10.11 | 1.117 | 0.357 |  |

**Table S2h.** *Model 8*, results of generalized additive model for wild bird infestation by nymphs *Amblyomma* ticks, with an adjusted R-squared of 0.053 and 15.5% deviance explained.

| Family: Quasibinomial | | | | | |
| --- | --- | --- | --- | --- | --- |
| Link function: Logit | | | | | |
| **Parametric coefficients:** | **Estimate** | **Std. Error** | ***t* value** | ***p*-value** |  |
| Intercept | -4.786 | 0.487 | -9.813 | <0.01 |  |
| Delta_temperature | -0.024 | -0.03 | -0.699 | 0.485 |  |
| Delta_precipitation | <0.001 | 0.001 | 0.397 | 0.691 |  |
| Elevation | <-0.001 | <0.001 | -0.246 | 0.806 |  |
| Habitat_natural | 0.622 | 0.430 | 1.448 | 0.149 |  |
| Habitat_semi-natural | 0.768 | 0.455 | 1.690 | 0.093 |  |
| Richness | -0.009 | 0.005 | -1.688 | 0.093 |  |
| **Approximate significance of smooth terms:** | **edf** | **Ref.df** | **F** | ***p*-value** |  |
| S ( Latitude) | 1 | 1.001 | 7.531 | 0.006 |  |

**Table S2i.** *Model 9,* results of generalized additive model for wild bird infestation by larvae *Amblyomma* ticks, with an adjusted R-squared of 0.182 and 34.7% deviance explained.

| Family: Quasibinomial | | | | | |
| --- | --- | --- | --- | --- | --- |
| Link function: Logit | | | | | |
| **Parametric coefficients:** | **Estimate** | **Std. Error** | ***t* value** | ***p*-value** |  |
| Intercept | -3.576 | 1.169 | -3.058 | 0.002 |  |
| Delta_temperature | 0.025 | 0.136 | 0.189 | 0.850 |  |
| Delta_precipitation | <0.001 | 0.002 | 0.339 | 0.735 |  |
| Elevation | <-0.001 | <0.001 | -0.562 | 0.574 |  |
| Habitat_natural | 0.762 | 0.625 | 0.674 | 0.501 |  |
| Habitat_semi-natural | 0.762 | 0.625 | 1.219 | 0.224 |  |
| Richness | -0.026 | 0.012 | -2.037 | 0.043 |  |
| **Approximate significance of smooth terms:** | **edf** | **Ref.df** | **F** | ***p*-value** |  |
| S (Longitude, Latitude) | 11.92 | 15.75 | 1.32 | 0.195 |  |

**Table S3.** Pairwise comparisons of habitat variables using Tukey's post hoc test in the nine generalized additive models across three habitat types: natural (N), semi-natural (S), and agricultural-semiurban (A). Significance codes: ** P < 0.01, * P < 0.05.

| ***t* ratio** | **Contrast** | | |
| --- | --- | --- | --- |
|  | **A–N** | **A–S** | **N–S** |
| Model 1 | 0.253 | -0.081 | -0.334 |
| Model 2 | 0.885 | -0.85 | -1.735** |
| Model 3 | <0.001 | -0.371 | -0.372 |
| Model 4 | 0.154 | -0.737 | -0.891** |
| Model 5 | -0.040 | -1.064 | -1.024* |
| Model 6 | -0.567 | -0.387 | 0.18 |
| Model 7 | -0.523 | -0.279 | 0.245 |
| Model 8 | -0.623 | -0.769 | -0.146 |
| Model 9 | -0.388 | -0.762 | -0.375 |

**
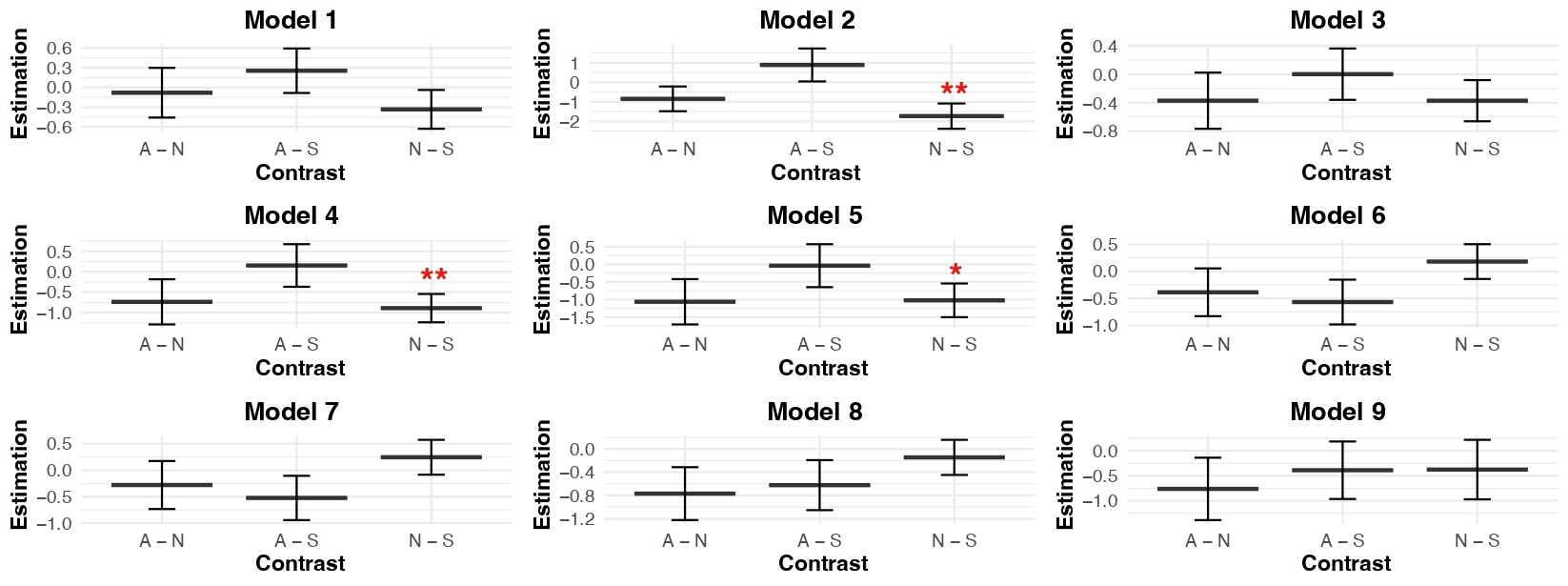
**

**Figure S1.** Error bar chart showing pairwise comparisons of habitat variables of bird infestation in the nine generalized additive models across three habitat types: natural (N), semi-natural (S), and agricultural-semiurban (A). Error bars represent 95% confidence intervals around the mean infestation proportion for each habitat type.
